# Synthetic Imaging Radar Data Generation in Various Clutter Environments Using Novel UWB Log-Periodic Antenna

**DOI:** 10.3390/s24247903

**Published:** 2024-12-11

**Authors:** Deepmala Trivedi, Gopal Singh Phartiyal, Ajeet Kumar, Dharmendra Singh

**Affiliations:** 1 Electronics and Communication Engineering, Indian Institute of Technology Roorkee, Roorkee 247667, India; dtrivedi@ec.iitr.ac.in; 2 School of Earth and Environment, University of Leeds, Leeds LS2 9JT, UK; g.s.phartiyal@leeds.ac.uk; 3 National Laboratory of Radar and Surveillance Systems (RaSS), National Inter-University Consortium for Telecommunications (CNIT), 56124 Pisa, Italy; ajeet.kumar@cnit.it

**Keywords:** through-the-wall imaging, through-the-foliage imaging, log-periodic antenna, range exponential power correction

## Abstract

In short-range microwave imaging, the collection of data in real environments for the purpose of developing techniques for target detection is very cumbersome. Simultaneously, to develop effective and efficient AI/ML-based techniques for target detection, a sufficiently large dataset is required. Therefore, to complement labor-intensive and tedious experimental data collected in a real cluttered environment, synthetic data generation via cost-efficient electromagnetic wave propagation simulations is explored in this article. To obtain realistic synthetic data, a 3-D model of an antenna, instead of a point source, is used to include the coupling effects between the antenna and the environment. A novel printed scalable ultra-wide band (UWB) log-periodic antenna with a tapered feed line is designed and incorporated in simulation models. The proposed antenna has a highly directional radiation pattern with considerable high gain (more than 6 dBi) on the entire bandwidth. Synthetic data are generated for two different applications, namely through-the-wall imaging (TWI) and through-the-foliage imaging (TFI). After the generation of synthetic data, clutter removal techniques are also explored, and results are analyzed in different scenarios. Post-analysis shows evidence that the proposed UWB log-periodic antenna-based synthetic imagery is suitable for use as an alternative dataset for TWI and TFI application development, especially in training machine learning models.

## 1. Introduction

The main aim of the microwave radar imaging system is to use microwave signals to sense and build an image of various targets for different environments/applications such as ground penetrating radar (GPR) [1], through-the-wall imaging (TWI) [2], through-the-foliage imaging (TFI) [3,4] and biomedical imaging [5]. Simulations and modeling are necessary steps toward developing any real system. A simulation is an approximate imitation of the operation of a process or system over time [6,7,8]. A simulator creates a virtual environment that acts as a mimic via a collection of software and hardware to complement labor-intensive experiments.

For target detection in electromagnetic simulations, Hertzian dipoles or lines of current are commonly employed to represent the antennas [9,10,11,12]. This primarily lowers the computational cost; however, mimicking realistic microwave radar systems and creating realistic data is still a challenging task [6,13]. In recent years, powerful computational resources have become available and accessible. Therefore, a 3-D model of the antenna can be included, instead of Hertzian dipoles or point sources, in the simulation scenario to obtain realistic synthetic data [7,14].

Antenna characteristics play an important role in microwave radar imaging (MRI) systems. The state-of-the-art antenna design for MRI systems focuses on achieving a wide bandwidth for better range resolution [15] and a compact size for portability (given an operating frequency band). Multi-purpose antenna designs applicable in different microwave imaging applications are desirable nowadays [16]. For example, TWI and GPR imaging systems operate in the 1–4 GHz (L and S) frequency band [17,18]; in contrast, lower frequency bands (VHF/UHF/L) are required for better penetration in TFI applications [19,20]. A considerable gain throughout the operating bandwidth is also desirable when designing MRI systems [18]. Finally, maintaining a directional radiation pattern throughout the bandwidth adds to the desirable antenna design characteristic [17,21].

To this end, a log-periodic antenna is investigated in this paper because of its stable radiation pattern throughout the bandwidth [22]. The log-periodic antenna is a frequency-independent antenna structure that consists of serval dipoles of exponentially varying length, and each one resonates at its own center frequency. All the dipoles of a log-periodic antenna are connected via a feeding system that impacts the input impedance significantly. For example, a typical microstrip feeding line has a constant width constraining impedance matching, resulting in a sub-optimal bandwidth [18]. A modified tapered feedline instead of a constant width feedline leads to a remarkable lower-frequency performance [23]. Therefore, in this study, the focus is to design a novel printed log-periodic antenna that has a compact size with a wide bandwidth using this modified feedline system. In this paper, this antenna design is simulated at two different dimension scales to be used in two different imaging radar applications, i.e., TFI and TWI.

In this paper, a simulation study is presented where synthetic imaging radar data are generated for different types of clutter environments (foliage and walls) using the novel antenna proposed here. For TWI, B-scan data are generated with two different considerations. In the first case, different wall types are set as scenarios with a single metal target. Four different wall types, namely brick, concrete, glass, and wood, are considered. In the second case, three different target placement scenarios are considered with a brick wall. For TFI, B-scan data are generated with three different considerations. In various cases, B scans are generated for different moisture content, different target placement, and different antenna orientations. After the generation of synthetic data, clutter removal techniques are also explored, and results are analyzed in different sceneries. Clutter removal techniques help separate clutter and target signals for a more accurate target prediction. The most common clutter removal techniques in recent years are based on subspace methods such as singular value decomposition (SVD), principal component analysis (PCA), robust principal component analysis (RPCA), and ICA (independent component analysis) [24,25,26,27]. In this article, SVD is used to reduce clutter. Along with SVD, range exponential power correction (REPC) is also explored, and all of the results are compared in this paper. Commercial software (CST Microwave Studio) suite-2021 is employed by implementing the finite-integration technique (FIT) for modeling and simulating TWI and TFI environments. To summarize, synthetic imagery is generated here using log-periodic antenna models, foliage and wall clutter environments, and clutter removal techniques for TWI and TFI applications.

The article is organized as follows: In Section 2, the overall methodology for synthetic radar data generation is discussed, and Section 3 further describes the antenna design and its characteristics. Section 4 is focused on the modeling of foliage environment for target detection and synthetic imaging radar data collection. The post-processing of synthetic data is explored in Section 5. Finally, we conclude the article in Section 6.

## 2. Methodology

**Figure 1:**
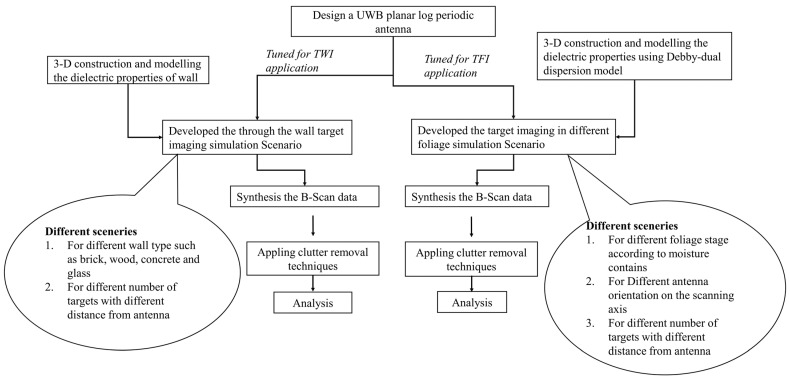
Methodology for generation of synthetic imaging radar data.

The methodology here details a process for generating synthetic imagery in various clutter environments using a UWB antenna. The complete methodology of this process is described in this section and graphically presented in Figure 1.

- Designing a high gain, stable radiation pattern and compact UWB printed planar antenna. Antenna design and analysis are discussed in Section 3.
- Scaling the design for two different applications, i.e., for TWI and TFI, via simply scaling the size of the antenna.
- Modeling different clutter environments for TWI and TFI applications. This step is described and discussed in detail in Section 4. After modeling the environments, synthetic data is generated using different clutter scenarios such as wall types, foliage moisture, number of targets, target positions, and antenna orientation.
- Lastly, post-processing to reduce the effect of clutters for the successful detection of targets. A detailed analysis of this step is discussed in Section 5.

## 3. Antenna Design

Usually, an antenna is designed to serve a specific application, and its size, gain, and bandwidth requirements are determined according to that application. However, any attempt to utilize this antenna design for a different application should fail. This is because other applications might need this antenna to operate at different conditions, such as different operating frequency ranges and different gain and bandwidth requirements. A multi-purpose antenna design could be used (with slight changes) for similar applications [16]. For example, most imaging radars work on the same principle and require similar antenna characteristics with few alterations. These alterations usually change the operating frequency range. A multi-purpose antenna designed to focus on such a family of applications is needed. To this end, a multi-purpose antenna design that can be used in different microwave imaging applications has been proposed in this paper. This study requires a multi-purpose antenna that works in multiple microwave imaging applications. These applications also require high gain, are highly directional, are operational in multi-frequency ranges, and have portable antennas. Among all candidates, a log-periodic antenna seems to be a good choice because of its wide bandwidth, high gain, and characteristics of directional radiation patterns [22].

**Figure 2:**
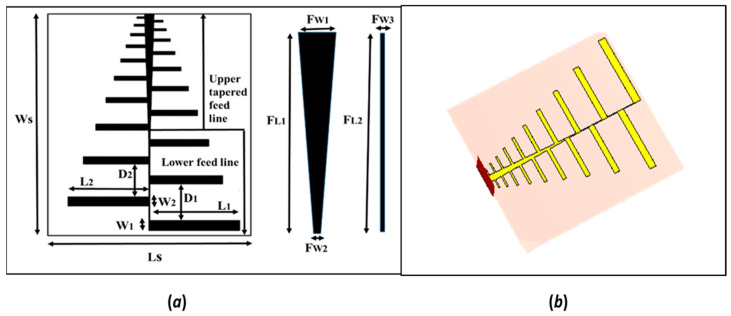
(**a**) Detailed geometry of the proposed log-periodic antenna (**b**) 3-D model of the proposed log-periodic antenna.

In this design, a total of 20 dipoles are utilized to create a log-periodic antenna with a scaling factor (α) and spacing factor set to 0.9. The substrate in the design is an FR-4 epoxy material having dielectric permittivity, ϵ_s_ = 4.3, and loss tangent, tanδ = 0.02. The feeding strategy used during the antenna design is a Microstrip feed line. The main benefit of a Microstrip feed line arrangement is that it makes the design flat, and the feed and patch can be grafted on the same substrate. Two vertically adjunct feed lines are used in which the lower feed line is a narrow rectangular structure that feeds the relatively longer dipole elements (lower five dipole elements as shown in Figure 2a (left)) while the upper feed line feeds the top 15 dipole elements. The upper feed line is tapered to improve the impendence mismatch from these shorter elements. The overall dimension of the antenna is 90 × 104 × 0.8 mm^3,^ which is suitable for portability. Figure 2b shows the 3-D model for the proposed antenna. The operating frequency of the resulting antenna is from 1.52 GHz to 5.42 GHz. This ultra-wide bandwidth (UWB) antenna is suitable for through-the-wall and underground imaging applications. This design is denoted by “Antenna_1” in this article from this point onwards. The aim here is to be able to obtain another antenna for a different purpose via a few design changes to the original. To this end, in the next step, the original design dimensions are scaled up by a factor of 2. The design is shown in Figure 2, and its dimensions are detailed in Table 1. Post-scaling, the operating frequency range of the new antenna shifted towards the lower side (0.7–2.7 GHz) while retaining all the other attributes. This antenna is now suitable for through-the-foliage imaging applications. This antenna is denoted by “Antenna_2” in the article from here onwards. Both antennas’ (Antenna_1 and Antenna_2) simulations (in CST studio) report highly directional radiation patterns with considerably high gain on the entire bandwidths.

**Figure 3:**
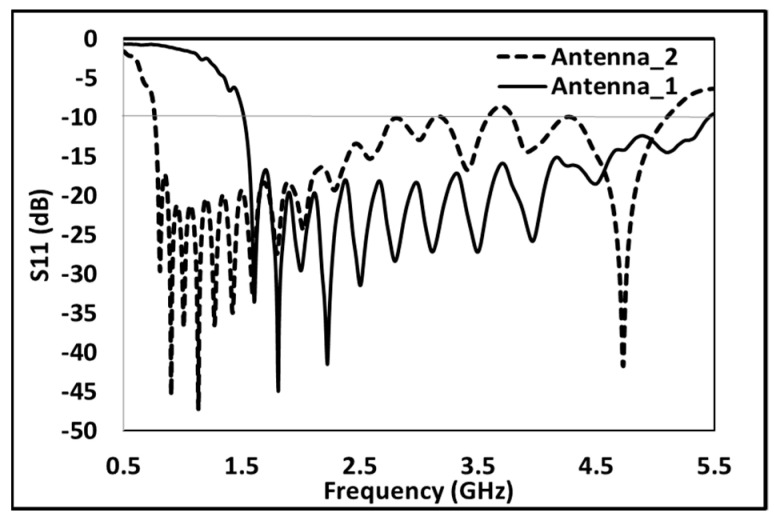
Reflection coefficient for proposed antennas.

**Figure 4:**
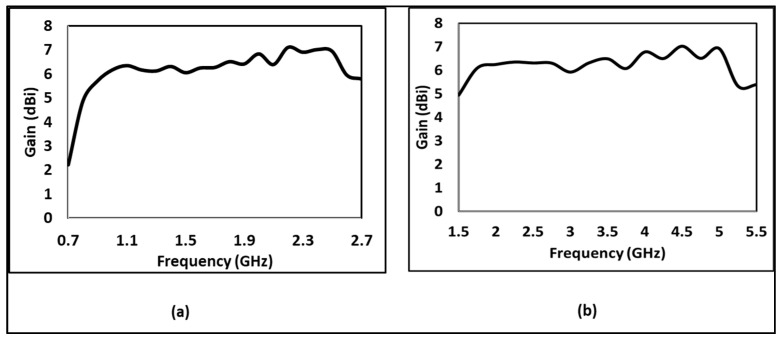
Gains of proposed antennas in a single direction (Theta = 90 degree and pi = 90 degree) (**a**) Antenna_2 (**b**) Antenna_1.

**Figure 5:**
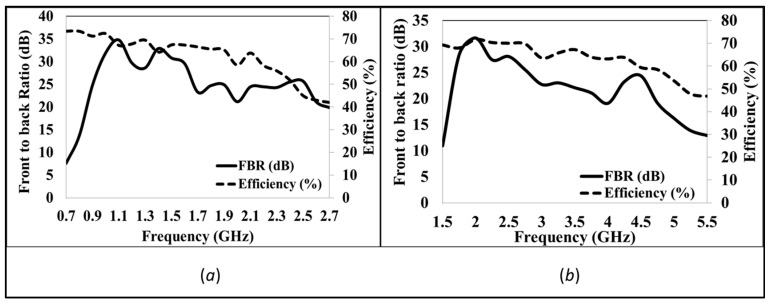
Front-to-back ratio (**a**) Antenna_2 (**b**) Antenna_1.

A performance analysis is performed for the proposed design. The return coefficient plotted against the frequency are shown in Figure 3. Also, the bandwidth is calculated to be below −10 dB power return. For Antenna_1, the achieved bandwidth is from 1.52 GHz to 5.42 GHz and is marked in a solid line in Figure 3. At the same time, Antenna_2 (scaled by scaling Antenna_1 by a factor of 2) achieves a bandwidth from 0.7 to 2.7 GHz and is marked by a dashed line in Figure 3. Figure 4a,b show the gain of Antenna_2 and Antenna_1 in a single direction (marked as Theta = 90 degrees and Phi = 90 degrees), respectively. From Figure 4a,b, it is evident that both antennas have achieved more than 6 dBi gain throughout corresponding bandwidths. Figure 5a,b show the front-to-back ratio (FBR) and efficiency for Antenna_2 and Antenna_1, respectively. The FBR range of Antenna_1 is 10.9 dB to 31.6 dB, whereas the FBR range of Antenna_2 is 7.6 dB to 34.75 dB. The average FBR across the bandwidth for both proposed antenna designs is above 20 dB. The average efficiency of both antennas across the bandwidth is above 60 percent.

**Figure 6:**
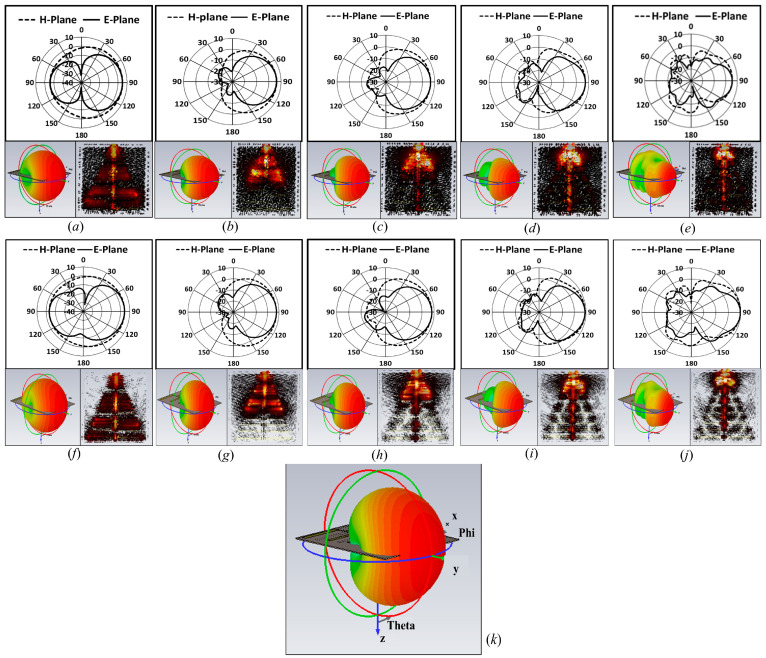
Far-field and current distribution of proposed antennas (**a**) at a frequency of 1.5 GHz for Antenna_1, (**b**) at a frequency of 2.5 GHz for Antenna_1, (**c**) at a frequency of 3.5 GHz for Antenna_1, (**d**) at a frequency of 4.5 GHz for Antenna_1, (**e**) at a frequency of 5.5 GHz for Antenna_1, (**f**) at a frequency of 0.7 GHz for Antenna_2, (**g**) at a frequency of 1.2 GHz for Antenna_2, (**h**) at a frequency of 1.7 GHz for Antenna_2, (**i**) at a frequency of 2.2 GHz for Antenna_2, and (**j**) at a frequency of 2.7 GHz for Antenna_2. (**k**) Annotations for reference.

Figure 6a–e show 1-D polar plots in E and H planes, 3-D polar plots, and surface current distribution at 1.5, 2.5, 3.5, 4.5, and 5.5 GHz, respectively, for Antenna_1. Similarly, Figure 6f–j show a 1-D polar plot in the E and H plan, a 3-D polar plot, and surface current distribution at 0.7, 1.2, 1.7, 2.2, and 2.7 GHz, respectively, for Antenna_2. Both antenna designs give a high direction and stable radiation pattern on the entire bandwidth, although the E-plane radiation field pattern gives a narrow beam width compared to the H-plane radiation field pattern. Figure 6 also presents the surface current distribution. From the analysis, it was concluded that, at a lower frequency of achieved bandwidth in both antennas, a strong surface current encompasses all elements. As the frequency is increased linearly, the strong surface current is limited to the thinner elements of the log-periodic antenna. These log-periodic antenna designs are suitable for microwave radar imaging applications like ground penetrating radar, through-the-wall imaging, and foliage penetrating radar due to the high bandwidth, considerably high gain, and stability of radiation patterns in a single direction in the entire bandwidth.

Antenna characteristics play an important role in microwave radar imaging systems, especially the antenna gain and bandwidth, which are important for imaging radar to achieve better penetration and fine resolution, respectively. Along with gain and bandwidth, stable radiation patterns using a compact-size antenna design are also desirable for microwave radar imaging systems. Table 2 shows a comparison of the novel antennas performance with other antennas that are used in similar applications. In comparison to other antennas that use 3-D structures, multilayers, or arrays to achieve operating conditions [28,29,30,31], the proposed antennas are relatively compact and have a planar log-periodic design. Antenna_1 also achieves an ultra-wide bandwidth of 4000 MHz, which is the highest among other antennas listed in Table 2 for TWI applications. On the other hand, Antenna_2 achieves an ultra-wide bandwidth of 2000 MHz, which is the highest among other antennas listed in Table 2 for TFI applications. Both antennas have more desirable characteristics such as high gain (>6 dB), high FBR, and a stable radiation pattern across the bandwidth. Antennas in [30] for TFI and [32] for TWI have higher gains than their proposed counterparts. However, this is achieved at the expense of larger sizes and design complexity. In summary, both Antenna_1 and Antenna_2 are suitable for use in the corresponding applications.

## 4. Environment Modeling and Simulation

Synthetic data have various advantages over real data, such as creating large datasets that include a vast verity of scenarios. With large datasets at hand, AI/ML-based techniques for post-processing and other applications can be easily developed [34]. Two sets of data are created in this section: a dataset for through-the-wall imaging applications and a dataset for through-the-foliage imaging applications. In both cases, two major steps are followed to create synthetic data. The first step is to model the environment, and the second step is to simulate the environment. The modeling environments are created and simulated in CST studio. The modeling and simulation of the two datasets are discussed separately here.

### 4.1. Modeling and Simulation: Through-the-Wall Imaging

**Figure 7:**
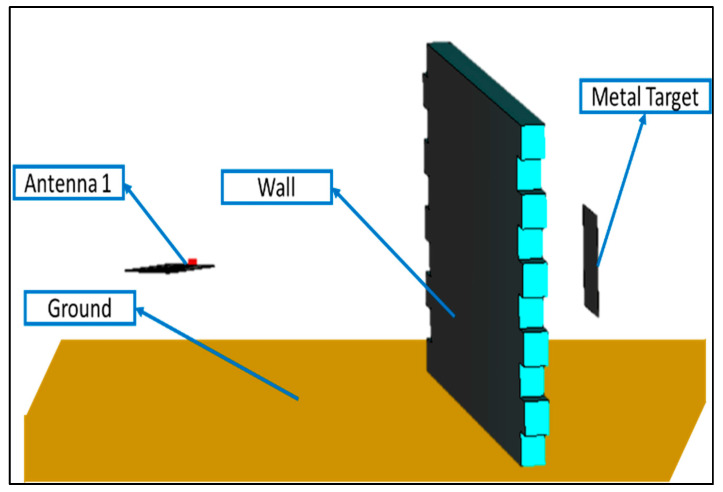
Through-the-wall imaging environment (wall with target and Antenna_1).

Through-the-wall imaging radars generally operate at frequencies ranging from 1 to 4 GHz, though this can be increased to achieve better range resolution. Based on this requirement, Antenna_1, that has an operating frequency band from 1.5 GHz to 5.5 GHz, was used. The achieved range resolution was 3.75 cm, which was suitable for this application. The dimensions of the wall considered as an obstruction here are 200 × 100 × 10 (width × height × depth) cm^3^, whereas four different plausible dielectric materials were considered to make the wall, i.e., brick, wood, glass, and concrete, as shown in Figure 7. These different material wall(s) stipulate different scenarios. These scenarios are listed in Table 3. Furthermore, multiple target scenarios were also stipulated. These are also listed in Table 3. Aluminum sheets with dimensions of 30 × 30 × 0.1 (height × width × thickness) cm^3^ were considered targets and were placed facing the wall. B-scans were generated and collected for each of these scenarios. These B-scans were processed further for target detection and are discussed in Section 5.1.

### 4.2. Foliage Environment

Unlike modeling a ‘wall’, modeling a foliage environment is more complex and involves the interaction of numerous different structures. Therefore, for the construction of a foliage environment, the Arbaro Tree Engine (ATE) [35] was used. ATE requires foliage parameters, such as position (spacing between individual plants), and plant parameters, such as height, species, and crown structure type. In this study, sugarcane crop was selected to act as foliage. A single sugarcane plant is composed of two basic structures, namely stem and leaf. The stem was constructed via a large number of cylinders of finite sizes, whereas the leaf was constructed via a large number of thin disks. A total of 12 sugarcane plants covering 90 × 60 (length × breadth) cm^2^ area were created to act as foliage. For modeling the sugarcane crop foliage environment using Arbaro Tree Engine (ATE), there are a few limitations. In this study, the sugarcane crop field had a density of 30 plants per square meter and all the plant sizes are assumed to be identical. This led to the limitation of placing 2 targets, at most, at the same range and different cross-range. Along with structural limitations, the dielectric constant of the whole sugarcane plant was set to a constant value to make the environment simpler. Due to these limitations, a big and diverse sugarcane crop field area was not studied here. In this paper, our focus is to propose a simulation model which can further extend to different scenarios. For modeling the foliage environment, this study followed the method proposed by the authors in [36]. Their method utilizes the “Dual Dispersion” (DD) dielectric mixing model for setting the dielectric properties of sugarcane plant structures [37]. Identical dielectric values for stem and leaves were used here. Through-the-foliage imaging radars generally operate VHF, UHF, and L-Band. Based on this requirement, Antenna_2, which has an operating frequency band ranging from 0.7 GHz to 2.7 GHz, was used here. The achieved range resolution was 7.5 cm, which was suitable for this application.

**Figure 8:**
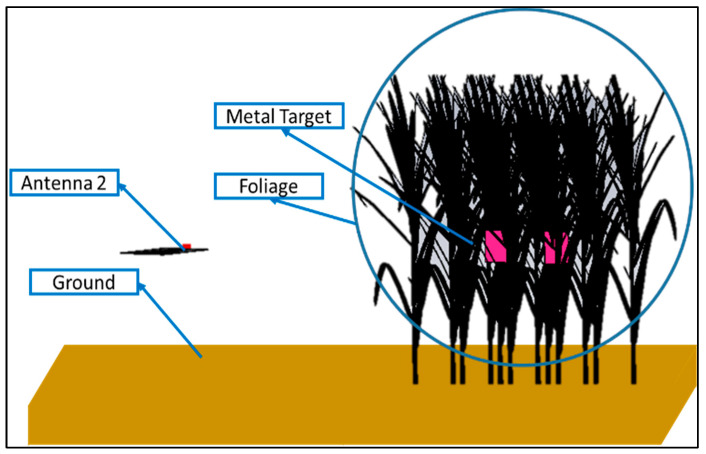
Foliage environment with target and Antenna_2.

Multiple through-the-foliage imaging simulation scenarios were created based on the moisture content of the foliage, antenna orientation with respect to ground, and number of targets. For the target, 10 × 10 × 0.1 cm^3^ sized aluminum sheets were placed inside the foliage. A foliage scenario is shown in Figure 8. A total of eight B-scans were obtained, and their details are listed in Table 4. After raw data generation in both scenarios, data analysis and post-processing were performed for target detection, as described in Section 5.2.

## 5. Post-Processing and Target Detection

The B-scans obtained from the through-the-wall and through-the-foliage imaging performed in the previous section needed to undergo post-processing steps such as time-gating and clutter reduction. Clutter removal techniques are very helpful in the separation of clutter and target signals and provide improved target detection. The most common categories of clutter removal techniques used in recent years are based on subspace methods, such as singular value decomposition (SVD), principal component analysis (PCA), robust principal component analysis (RPCA), and ICA (independent component analysis) [24]. In this study, SVD was explored for clutter reduction. SVD is a very effective and stable factorization technique for diagonalizing matrices because the system can be decomposed into a set of linearly independent components, each with an associated weight. Let us consider a raw B-scan (with target and clutter present) represented by the matrix $A_{ij}$ with dimensions N X M; N ≥ M and i = 1, 2, …, N; j = 1, 2, …, M, where the indices i and j indicate time and the antenna position, respectively. The mean subtraction is performed by calculating the mean vector of the B-scan and then subtracting it from each individual A-scan, as expressed in Equation (1).

(1) $$X_{ij}=A_{ij}-\frac{1}{N}\sum_{j=1}^{M}A_{ij}$$

With the SVD method, the matrix *X*_ij_ can be represented as Equation (2).

X = USV^T^ (2)

where U and V are (N × N) and (M × M) unitary matrices, respectively, and S = *diag*(σ_1_, σ_2_, …, σ_r_) with σ_1_ ≥ σ_2_ ⋯ ≥ σ_r_ ≥ 0. Further, the matrix X_ij_ can be decomposed into subspaces, as shown in Equations (3) and (4).

(3) $$X=\sum_{i=1}^{M}\sigma_{i}\mu_{i}v_{i}$$

or

(4) $$X=N_{1}+N_{2}+-----+N_{N}$$

where N_i_ are the matrices of the same dimensions as X, which are called its modes or the Eigen image of X. This technique is generally used in data compression and is very useful for filtering out noise.

In the range power correction exponential (RPCE), each time stamp of the signal from one ‘antenna position’, i.e., a column of the B-scan*,* is multiplied by the square of the range bin where ΔR is range resolution.

(5) Range bin=[ΔR    2ΔR    3ΔR ………………NΔR]

Y_i_ = B_i_ (.) Rangebin^2^ (6)

where (.) is element to element multiplication. Y is the B-scan after applying RPCE.

### 5.1. Target Detection in Through-the-Wall Imagery

**Figure 9:**
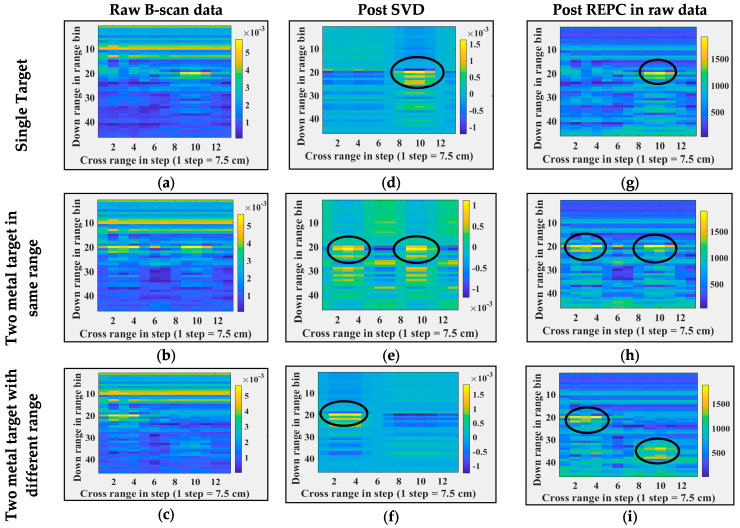
Through-the-wall imaging and post-processing at different target(s) locations. (**a**–**c**) Raw B-scans, (**d**–**f**) B-scans post SVD operation, (**g**–**i**) B-scans post REPC operation, (**a**,**d**,**g**) for a single target, (**b**,**e**,**h**) for two targets at the same range and different cross-range, and (**c**,**f**,**i**) for two targets at different range and cross range. Black circles in each image represent the targets’ locations.

**Figure 10:**
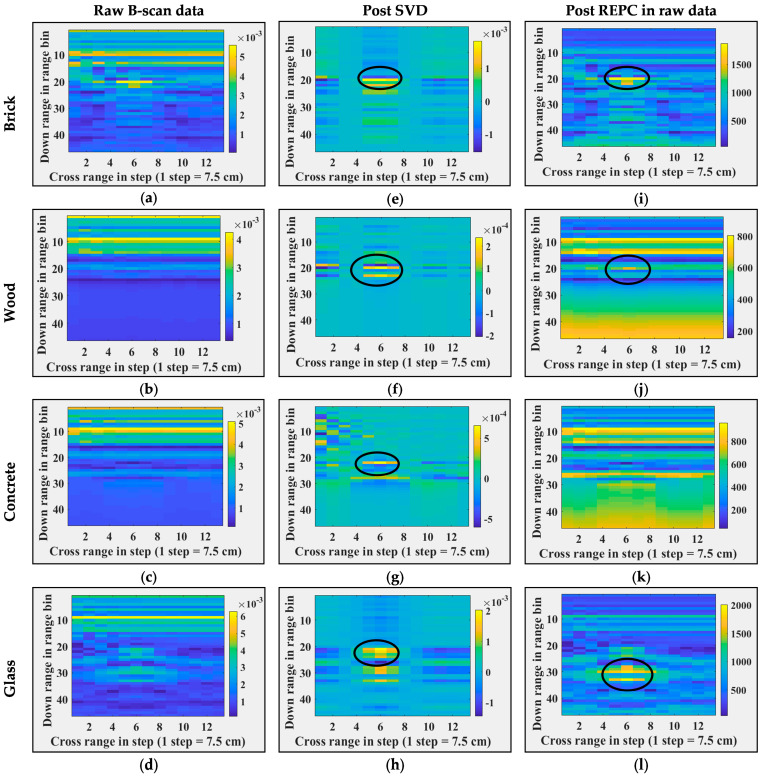
Through-the-wall imaging and post processing with different walls. (**a**–**d**) Raw B-scans, (**e**–**h**) B-scans post SVD operation, (**i**–**l**) B-scans post REPC operation, (**a**,**e**,**i**) for a brick wall, (**b**,**f**,**j**) for a wood wall, (**c**,**g**,**k**) for a concrete wall, and (**d**,**h**,**l**) for a glass wall. Black circles represent the targets locations.

**Figure 11:**
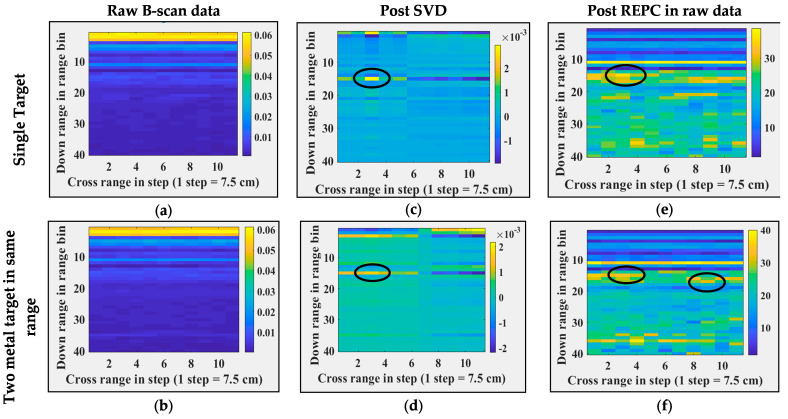
Real-time through-the-wall imaging and post-processing. (**a**,**b**) Raw B-scans, (**c**,**d**) B-scans post SVD operation, (**e**,**f**) B-scans post REPC operation, (**a**,**c**,**e**) for a single target, and (**b**,**d**,**f**) for two targets at the same range and different cross-range. Black circles represent the targets locations.

First, all seven B-scans are time-gated based on the position of antenna-free space mismatch range bins in the B-scans. Next, singular value decomposition (SVD) and RPCE are applied to these time-gated B-sans. Figure 9 and Figure 10 illustrate images post each processing step, i.e., column 1 corresponds to time-gated B-scans, column 2 corresponds to B-scans post SVD, and column 3 corresponds to B-scans post RPCE. Figure 9 shows the results for when the target position and target count are varied, and Figure 10 shows the results for when the wall dielectric (wall material) is varied. Figure 9a shows a B-scan with a single target placed at 150 cm from the antenna (range bin), Figure 9b shows a B-scan with two targets placed at the same range bin (150 cm) but at different cross-range bins (bin 4, and bin 10), and Figure 9c shows a B-scan with two targets placed at different range and cross-range bins. These details are also listed in Table 3 for better understanding. Post SVD, single targets and multiple targets placed at the same range bin were detected successfully, as shown in Figure 9d,e. The SVD approach is thus able to separate the target from the wall (clutter). Post SVD, the first Eigenvector corresponds to the wall as it had a maximum variance, and the second Eigenvector corresponds to the target(s). Figure 9d,e show B-scan projections corresponding to the second Eigenvector. In the case of multiple targets placed at different ranges and cross-range bins, SVD was only able to detect targets with variance higher than noise, i.e., the target close to the antenna, and was unable to detect the one farther away. Results post SVD, in this case, are shown in Figure 9f. Figure 9g–i show results post RPCE (applied to time-gated B-scans). It is evident from the results that RPCE was able to extract the targets (single or multiple) successfully. This is due to the correction in the backscatter response performed via RPCE. In summary, the RPCE method is successful in extracting multiple targets in through-the-wall imaging scenarios, whereas the SVD method fails when multiple targets are present at different ranges. Further, Figure 10 illustrates a second scenario, where B-scans were observed with a single target (metal) and different wall types (Brick, Wood, Glass, and Concrete). Further details on these experiments are mentioned in Table 3. In Figure 10, Column 1 shows raw B-scans after time gating. SVD is applied to these B-scans, and Figure 10, column 2, corresponds to the results post SVD. These results show that SVD successfully detected the target in each wall-type scenario. Figure 10, column 3 corresponds to the results post RPCE. The RPCE successfully detected targets in each wall type except for concrete. Both methods indicated the presence of targets behind the wall. In summary, post-processing indicated the successful observations of B-scans and the presence of targets in them. To evaluate the performance on real scenarios, similar target detection techniques applied on real experimental data of through-the-wall imaging and the corresponding results are shown in Figure 11. Column 1, column 2, and column 3 show the raw B-scan data, data post SVD applied directly on raw data, and data post REPC applied directly on raw data, respectively. The analogous performance of target detection techniques on both real and synthetic TWI data indicates the good credibility and integrity of synthetic data. Therefore, the synthetic data generated using Antenna_1 are potentially useful data and can be utilized in the development of other clutter removal and target detection methods. It is important to note here that these experiments do not reflect the ability of SVD and REPC to detect targets in clutter environments; instead, they reflect the credibility of the synthetic data to be used for algorithm development if real data are not available.

### 5.2. Target Detection in Through-the-Foliage Imagery

**Figure 12:**
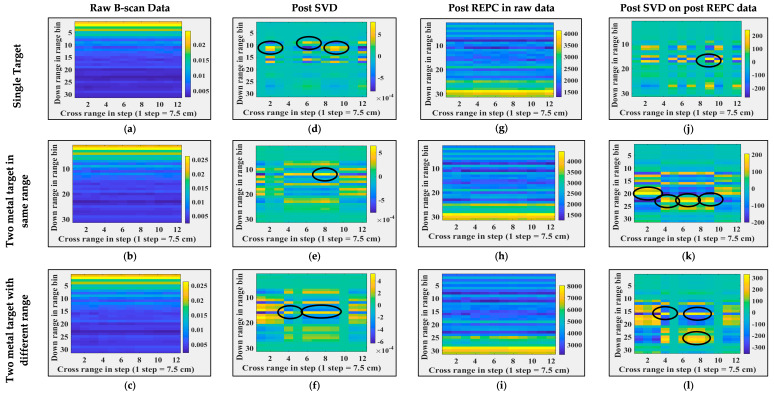
Foliage penetrating radar imaging and post-processing when the antenna was vertically oriented. (**a**–**c**) Raw B-scans, (**d**–**f**) B-scans post SVD operation, (**g**–**i**) B-scans post REPC operation, (**j**–**l**) B-scans post SVD operation on post REPC data, (**a**,**d**,**g**,**j**) for a single target, (**b**,**e**,**h**,**k**) for two targets at the same range and different cross-range, and (**c**,**f**,**i**,**l**) for two targets at different range and cross-range. Black circles represent the targets locations.

**Figure 13:**
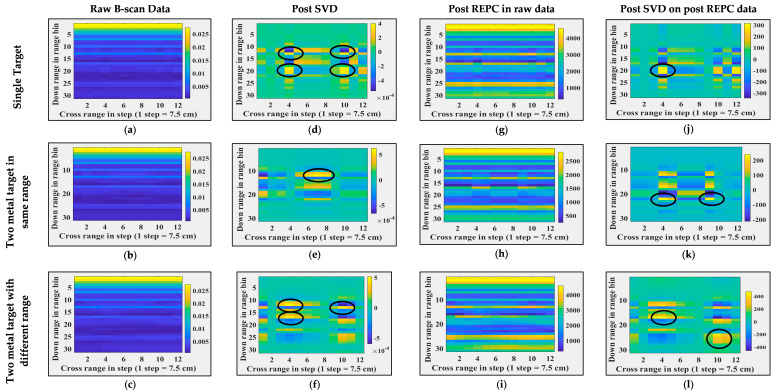
Foliage penetrating radar imaging and post-processing when antenna is horizontally oriented. (**a**–**c**) Raw B-scans, (**d**–**f**) B-scans post SVD operation, (**g**–**i**) B-scans post REPC operation, (**j**–**l**) B-scans post SVD operation on post REPC data, (**a**,**d**,**g**,**j**) for a single target, (**b**,**e**,**h**,**k**) for two targets at the same range and different cross-range, and (**c**,**f**,**i**,**l**) for two targets at different range and cross-range. Black circles represent the targets’ locations.

**Figure 14:**
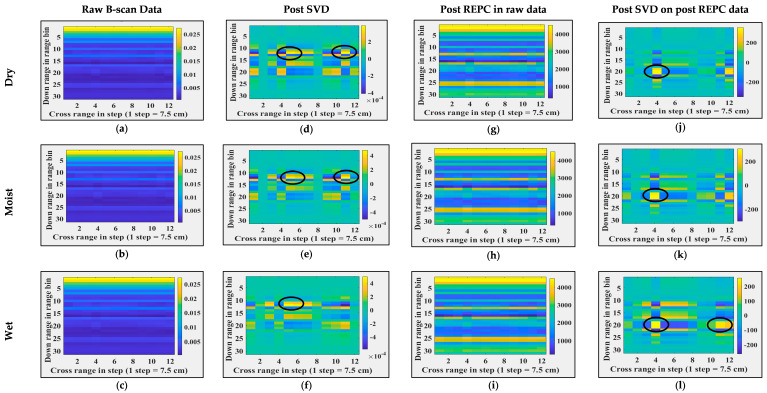
Foliage penetrating radar imaging and post-processing for different moisture content. (**a**–**c**) Raw B-scans, (**d**–**f**) B-scans post SVD operation, (**g**–**i**) B-scans post REPC operation, (**j**–**l**) B-scans post SVD operation on post REPC data, (**a**,**d**,**g**,**j**) for dry foliage, (**b**,**e**,**h**,**k**) for moist foliage, and (**c**,**f**,**i**,**l**) for wet foliage. Black circles represent the targets locations.

A total of eight B-scans were collected in this case. These B-scans were time-gated in a manner similar to those mentioned in Section 5.1. Figure 12 illustrates the B-scans and their post-processing results when antenna orientation was set to ‘vertical’ and target count and position were varied. Figure 13 illustrates the B-scans and their post-processing results when antenna orientation was set to ‘horizontal’ and target count and position were varied. Figure 14 illustrates the B-scans and their post-processing results when foliage moisture content were varied. In Figure 12, Figure 13 and Figure 14, column 1 shows time-gated B-scans, column 2 shows results post SVD, column 3 shows result post RPCE, and column 4 shows results post (RPCE+SVD). The following observations that are similar in all of the cases can be made: (i) presence of relatively heavy clutter, and (ii) presence of target(s). In the cases where the antenna orientation was vertical (see Figure 12), the targets were detected with the RPCE+SVD method. The SVD+RPCE also provided false alarms (see Figure 12k,l. Alternatively, where the antenna orientation was horizontal (see Figure 13), the targets were detected with the SVD method (see Figure 13d) and RPCE+SVD method (see Figure 13j). However, the clutter was more suppressed in RPCE+SVD than in SVD only. When comparing results with different antenna orientations, i.e., vertical and horizontal, less clutter is observed when imaging was performed with a horizontal antenna orientation. This observation aligns with similar observations in other studies [38]. Analysis on changes in foliage moisture content was carried out and is shown in Figure 14. As we increased the moisture content of the foliage, the backscattering from the foliage was more prominent, as shown in Figure 14, column 4.

**Figure 15:**
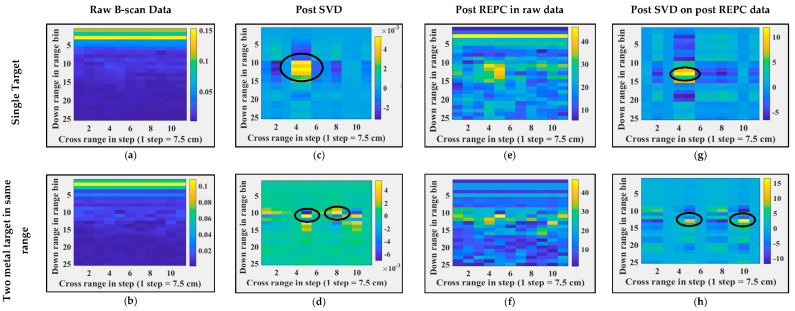
Real-time foliage penetrating radar imaging and post-processing when the antenna is horizontally oriented. (**a**,**b**) Raw B-scans, (**c**,**d**) B-scans post SVD operation, (**e**,**f**) B-scans post REPC operation, (**g**,**h**) B-scans post SVD operation on post REPC data, (**a**,**c**,**e**,**g**) for a single target, and (**b**,**d**,**f**,**h**) for two targets at the same range and different cross-range. Black circles represent the targets’ locations.

Both SVD and REPC target detection techniques are applied to real experimental data of through-the-foliage imaging, and the corresponding results are shown in Figure 15. Column 1, column 2, column 3, and column 4 show the raw B-scan data, data post SVD applied directly on raw data, post REPC, and post SVD on post-REPC data, respectively. The analogous response of SVD and REPC on both real and synthetic through-the-foliage data indicates the good credibility and integrity of synthetic TFI data. Therefore, the synthetic data generated using Antenna_2 are potentially useful data and can be utilized in the development of other clutter removal and target detection methods. Similar to experiments in TWI (refer to Section 5.1), it is important to note here that these experiments do not reflect the ability of SVD and REPC to detect targets in a clutter environment; instead, they reflect the credibility of the synthetic data to be used for algorithm development if real data are not available.

In summary, a few important takeaways from these experiments are as follows.

- The proposed antenna design is suitable for use in generation of synthetic TWI and TFI data.
- The clutter models are a simpler approximation of a real-world clutter environment.
- The synthetic data generated in the case of TWI and TFI are credible and could be used in development of novel clutter removal or target detection algorithms.

## 6. Conclusions

In this paper, synthetic data were successfully generated for short-range TFI and TWI applications using a scalable novel log-periodic antenna to understand the backscattering behaviour of hidden targets in different clutter environments. For this purpose, a scalable novel log-periodic antenna was designed to be suitable for use in TWI and TFI applications simultaneously. Two antennas, i.e., Antenna_1 and Antenna_2, were designed and utilized for the generation of synthetic data for TWI and TFI applications. Scalability was achieved by simply scaling the size (dimensions) of the original design. Antenna_1’s bandwidth extends from 1.5 GHz to 5.5 GHz with a 6 dBi gain throughout the bandwidth and is suitable for TWI applications. On the other hand, Antenna_2 was designed to operate from 0.7 to 2.7 GHz with 6 dBi gain throughout the bandwidth, which makes it suitable for use in TFI applications. Radiation patterns for both Antenna_1 and Antenna_2 are stable throughout their corresponding bandwidths. The average front-to-back ratio, or FBR, achieved was higher than 20 dB throughout the bandwidth for both the antennas. In addition to these features, both antennas are compact in size. Numerous B-scans were generated for both TWI and TFI, encapsulating various clutter scenarios. These scenarios include varying wall types, foliage moisture, number of targets, target positions, and antenna orientation. After the generation of synthetic data, clutter removal techniques were also applied, and results were analyzed. Post analysis, it is evident that both antenna designs are a good choice for radar imaging system simulations and data generation. In summary, the synthetic data generation strategy for TWI and TFI is reliable and can be used at-scale to generate loads of TWI and TFI datasets. These datasets can further be a good source for development of more efficient clutter removal or target detection methodologies in TWI and TFI.

## Figures and Tables

**Table 1:** Parameter details of the proposed log-periodic antennas.

Parameters	Antenna_1 (mm)	Antenna_2 (mm)
L1	40	80
L2	36	72
W1	4.5	9
W2	4.05	8.1
D1	7	14
D2	6.3	12.6
LS	104	208
Fw1	4	8
Fw2	0.5	1
Fw3	0.5	1
d	0.8	1.6
FL1	59	118
FL2	42.5	85
WS	90	180

**Table 2:** Performance of the proposed antenna in comparison with the other reported works that are used in similar applications.

Reference	Application	Structure Type	Size	Gain (dBi)	Bandwidth (MHz)
[28]	L-band foliage penetration (FOPEN)	2 dielectric layers and 3 metallic layers	41λ × 0.41λ × 0.0038λ	>2.31	550
[29]	UHF band foliage penetration (FOPEN)	3-D discone structure	Cylindrical volume with 600 mm (D) and 700 mm (H)	>0.5	20 and 16
[30]	UHF band foliage penetration (FOPEN)	Planar array structure	1200 × 2438 mm^2^	>7	500
[32]	Not specified	3-D discone structure	Cylindrical volume with 0.227 λ0 (D) and 0.096 λ0 (H)	>0.2	2280
[33]	S-band TWI	Vivaldi antenna	58.9 × 48 mm^2^	5 to 6	1240
[31]	S-band TWI	1 × 8 Vivaldi antenna array	480 × 210 mm^2^	>12	3000
Antenna_2	UHF to L band TFI	Planar log-periodic	180 × 208 mm^2^	>2.31	2000
Antenna_1	L to S-band TWI	Planar log-periodic	90 × 104 mm^2^	>0.5	4000

**Table 3:** Data collection details in the through-the-wall imaging environment.

Sr. No.	Type of Wall	Number of Targets	Distance of Target from the Antenna in cm	Cross Range Bin
1	Brick	1	150	10
2	Brick	2	150, 150	4, 10
3.	Brick	2	150, 200	4, 10
4.	Brick	1	150	6
5.	Wood	1	150	6
6.	Glass	1	150	6
7.	Concrete	1	150	6

**Table 4:** Data collection details in the foliage environment.

Sr. No.	Dielectric of Foliage	Antenna Orientation	Number of Targets	Distance of Target from Antenna in cm	Cross Range Bin
1	15 + 6i	Horizontally placed	1	150	4
2	15 + 6i	Horizontally placed	2	150, 150	4, 9
3.	15 + 6i	Horizontally placed	2	150, 175	4, 9
4.	15 + 6i	Vertically placed	1	150	4
5.	15 + 6i	Vertically placed	2	150, 150	4, 9
6.	15 + 6i	Vertically placed	2	150, 175	4, 9
7.	10 + 4i	Horizontally placed	1	150	4
8.	20 + 8i	Horizontally placed	1	150	4

## Data Availability

The original contributions presented in this study are included in the article. Further inquiries can be directed to the corresponding author.
